# Protocol to improve isoform-level quantification of low-abundance transcripts via STALARD pre-amplification

**DOI:** 10.1016/j.xpro.2026.104792

**Published:** 2026-08-25

**Authors:** Daesong Jeong, Chulmin Park, Ilha Lee

**Affiliations:** 1 Laboratory of Plant Developmental Genetics, School of Biological Sciences, Seoul National University, Seoul 08826, Korea; 2 Research Center for Plant Plasticity, School of Biological Sciences, Seoul National University, Seoul 08826, Korea

**Keywords:** Genetics, Genomics, Sequencing, Plant sciences, Molecular Biology, Gene Expression

## Abstract

STALARD (selective target amplification for low-abundance RNA detection) enables isoform-level quantification of low-abundance RNAs using conventional laboratory equipment. Here, we describe steps for RNA isolation, primer design, reverse transcription, selective target amplification, and downstream analysis. The protocol couples selective pre-amplification with a quantitative reverse-transcription PCR (RT-qPCR) readout and optional nanopore sequencing. Using 1 μg input RNA and 12 pre-amplification cycles, STALARD reduces Cq values by approximately 10–12 cycles, bringing the target into a reliably quantifiable range.

For complete details on the use and execution of this protocol, please refer to Jeong et al.^1^

## Highlights

• Instructions for isolating total RNA and designing gene-specific oligo(dT) primers
• Steps for cDNA synthesis with GSoligo(dT) to append a shared gene-specific sequence
• Procedures for selective single-primer pre-amplification with reduced primer-pair bias
• Guidance on RT-qPCR analysis and optional UMI-based nanopore isoform quantification

## Abstract (editor highlights)

Publisher’s note: Undertaking any experimental protocol requires adherence to local institutional guidelines for laboratory safety and ethics.

## Graphical abstract

**Figure:**
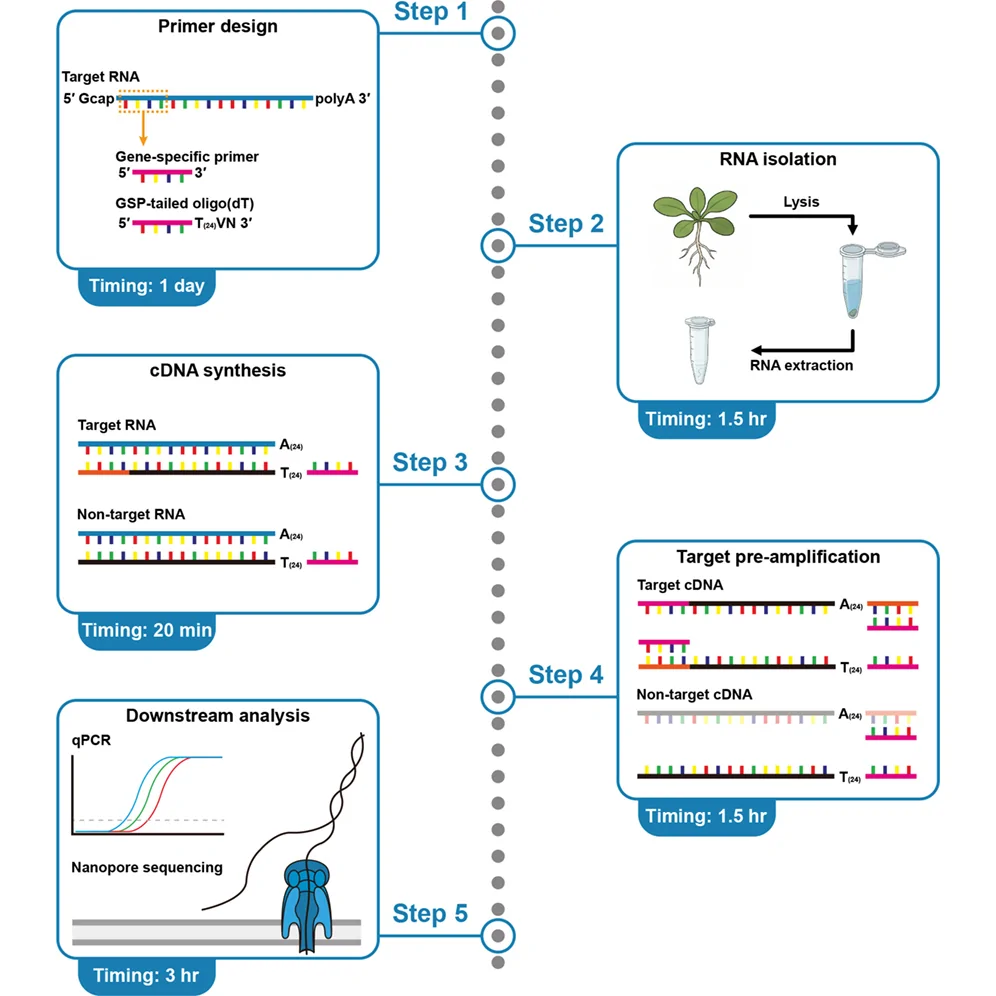

## Before you begin

STALARD is a targeted pre-amplification strategy designed to expand the reliable quantification range of conventional RT-qPCR into the low-abundance window (Cq > 30).^2^^,^^3^ This protocol provides a step-by-step workflow for sensitive and reproducible quantification of low-abundance RNA isoforms. *Arabidopsis* seedlings are used as the validation system, with the low-abundance transcript *VIN3* and the alternatively spliced transcripts of *FLM*, *MAF2*, *EIN4*, *ATX2*, and *COOLAIR* serving as representative targets (as described in Jeong et al.^1^). The protocol can be adapted to other polyadenylated transcripts, provided that the 5′-end sequence of the target is known. It is intended for researchers experienced in molecular biology techniques, including qPCR, but who may not have access to specialized platforms such as digital PCR. The workflow consists of selective single-primer target pre-amplification followed by one of two readout options: (i) isoform-specific RT-qPCR for relative quantification (the core readout), and (ii) optional nanopore amplicon sequencing with UMIs for molecule-level isoform counting. The RT-qPCR readout is sufficient for most applications; nanopore sequencing is an optional extension.

### Innovation

STALARD extends the reliable RT-qPCR quantification range by reducing Cq values by approximately 10–12 cycles while preserving relative isoform abundances. The key innovation is a single-primer amplification design: the gene-specific primer (GSP) is selected from a known 5′-end sequence shared among the targeted isoforms and appended to an oligo(dT) tract. Reverse transcription generates first-strand cDNA carrying the primer-derived GSP sequence at its 5′ end and the complementary target-derived sequence (cGSP) at its 3′ end. These terminal sequences enable subsequent pre-amplification with a single GSP across all targeted isoforms sharing that 5′ sequence. Using a common GSP rather than isoform-specific primer pairs minimizes differential amplification bias and helps preserve the original relative isoform abundances. Isoform-level resolution is achieved at the readout step, either by isoform-specific RT-qPCR primers or, optionally, by nanopore molecule-level counting with UMIs.

### Primer design guidelines

Primer design is critical for STALARD performance. The GSoligo(dT) primer carries a gene-specific sequence identical to the transcript’s 5′-end sequence (GSP), enabling selective amplification. The following principles are recommended.

1. Select a 20–25 bp sequence from the annotated 5′-region of the transcript (T substituted for U).
2. Design the primer for a melting temperature (Tm) of 60°C–62°C and a GC content of 40%–60%.
3. Avoid secondary structures, hairpins, or self-dimers.
4. Separately, design isoform-specific primer sets for qPCR-based detection of individual isoforms (distinct from the GSP above) using primer-design software such as Primer3 or Primer-BLAST, targeting an amplicon of 70–150 bp.

For specific primer sequences used in the validation experiments, refer to Jeong et al.,^1^ Supplementary Information.

***Optional:*** UMI incorporation for nanopore-based quantitative comparison.

For applications requiring molecule-level counting via nanopore sequencing, a unique molecular identifier (UMI) of 10–12 random bases can be incorporated into the GSoligo(dT) primer between the GSP sequence and the oligo(dT) tract: 5′-GSP-N_10-12_-dT_24_VN-3′.

The UMI is incorporated into each cDNA molecule during reverse transcription and is preserved through subsequent amplification, enabling collapse of PCR duplicates during downstream nanopore data analysis. This addition does not alter qPCR-based results.

## Key resources table

**Table:** 

REAGENT or RESOURCE	SOURCE	IDENTIFIER
**Chemicals, peptides, and recombinant proteins**		

NucleoZOL	Macherey-Nagel	Cat# MN740404
SeqAmp DNA polymerase	Takara	Cat# 638504
AMPure XP beads	Beckman Coulter	Cat# A63881
EvaGreen qPCR Master Mix	Biofact	Cat# DQ376-40h
NEB Blunt/TA Ligase Master Mix	New England Biolabs	Cat# M0367

**Critical commercial assays**		

HiScript IV 1st Strand cDNA Synthesis Kit	Vazyme	Cat# R412-01
Native Barcoding Kit 24 V14	Oxford Nanopore	Cat# SQK-NBD114.24
NEBNext Ultra II End Repair/dA-Tailing Module	New England Biolabs	Cat# E7546
NEBNext Quick Ligation Module	New England Biolabs	Cat# E6056
Qubit™ dsDNA HS Assay Kit	Thermo Fisher Scientific	Cat# Q32851

**Oligonucleotides**		

*UBC21* primer qPCR Forward: CTGCGACTCAGGGAATCTTCTAA qPCR reverse: TTGTGCCATTGAATTGAACCC	Custom synthesis	User-defined
*VIN3* primer GSP: CTTCTGAATCTTGTGTCTCGTCCTC GSoligo(dT)24VN: CTTCTGAATCTTGTGTCTCGTCCTCTTTTTTTTTTTTTTTTTTTTTTTTVN qPCR Forward: GTTTCAGGACAAGGTGACAAG qPCR reverse: GTAGAAGAAGACGGCTCCTCG	Custom synthesis	User-defined
*FLM* primer GSP: GTCACTTTCTCCAAACGACGCAATG GSoligo(dT)24VN: GTCACTTTCTCCAAACGACGCAATGTTTTTTTTTTTTTTTTTTTTTTTTVN Common qPCR Forward: TCCGGAAAACTCTATGACTCTTC *FLM-β* qPCR reverse: TCTGAATTTTTTCTTCAAGATCTAAGGC *FLM-δ* qPCR reverse: TCTGAATTTTTTCTTCAAGATCGAGTTC	Custom synthesis	User-defined
*ATX2* primer GSP: AGCGTTTACTCCGTTTCCTCTTCCT GSoligo(dT)24VN: AGCGTTTACTCCGTTTCCTCTTCCTTTTTTTTTTTTTTTTTTTTTTTTTVN *ATX2.1* qPCR Forward: ACAAGAGGCACATTACAATCTTTG *ATX2.1* qPCR Reverse: CCATCGTGTATAAAGAACCTGATCA *ATX2.3* qPCR Forward: AGAGTTTGTTCTTTGCAGCTACT *ATX2.3* qPCR Reverse: AGTCATCTTCTTCAATGGAGATAAG	Custom synthesis	User-defined

**Other**		

Mastercycler® nexus X2 thermal cycler	Eppendorf	Cat# 6337000019
CFX96 Touch Real-Time PCR Detection System	Bio-Rad	Cat# 185-5195
Flow Cell (R10.4.1)	Oxford Nanopore	Cat# FLO-MIN114
MinION sequencer (Mk1B)	Oxford Nanopore	Cat# MIN-101B
TissueLyser II (or equivalent bead homogenizer)	Qiagen	Cat# 85300
MagListo™ Magnetic Separation Rack	Bioneer	Cat# TM-1011
Mylab Inteli-mixer SLRM-2M	SeouLin Bioscience	Cat# SLRM-N1.5
Vortex-Genie 2	Scientific Industries	Cat# SI-0236
Refrigerated Microcentrifuge	Eppendorf	Cat# 5425R
Qubit 4 Fluorometer	Thermo Fisher Scientific	Cat# Q33238
Certified chemical fume hood	Local facility	N/A
3 mm Diameter Stainless Steel Bead	N/A	N/A
DNA LoBind Tube (Nuclease-free)	Eppendorf	Cat# 0030108051
DEPC-treated D.W. (Nuclease-free)	In-house	N/A
8-strip PCR Tube (Autoclaved)	Taeshin Bioscience	Cat# BL-0210DS
EXCELL AG 1250 μL Refill Tip (Nuclease-free)	LPS Solution	Cat# STAG-1250-RTS
EXCELL AG 200 μL Refill Tip (Nuclease-free)	LPS Solution	Cat# STAG-200-RTS

## Materials and equipment

Key reagents and instruments are summarized in the key resources table. Recipes are provided below; additional notes are included within the step-by-step section as needed.

**Note on polymerase choice.**

This protocol uses SeqAmp DNA polymerase (Takara, Cat# 638504), consistent with Jeong et al.^1^ Other low-bias, high-fidelity DNA polymerases, such as RepliQa HiFi ToughMix (Quantabio, Cat# 95200), may be substituted, particularly when downstream nanopore amplicon sequencing is planned and reduced amplification bias during long-range PCR is beneficial. Users adopting alternative polymerases should validate cycle-dependent specificity by melt curve analysis before routine use.

### Mastermix recipes

**Table:** RT master mix (Step 24)

Reagent	Volume	Stock concentration	Final concentration
HiScript IV RT SuperMix	5 μL	4×	1×
GSoligo(dT)	1 μL	50 μM	2.5 μM
Nuclease-free DW	4 μL	–	Total 10 μL

Prepare fresh immediately before use, keep on ice (0°C–4°C), and use within 1 h; do not store.

**Table:** Pre-amplification master mix (Step 30)

Reagent	Volume	Stock concentration	Final concentration
SeqAmp PCR buffer	25 μL	2×	1×
SeqAmp DNA polymerase	1 μL	62.5 U	1.25 U
GSP	1 μL	10 μM	0.2 μM
Nuclease-free DW	3 μL	–	–

Prepare fresh immediately before use, keep on ice (0°C–4°C), and use within 1 h; do not store.

**Table:** qPCR primer set (Step 47)

Reagent	Volume	Stock concentration	Final concentration
Forward primer	5 μL	100 μM	5 μM
Reverse primer	5 μL	100 μM	5 μM
Nuclease-free DW	90 μL	–	Total 100 μL

Store at 4°C for up to 1 week.

**Table:** End-prep master mix (Step 53)

Reagent	Volume	Stock concentration	Final concentration
Ultra II End-prep Reaction Buffer	1.75 μL	8.57×	1×
Ultra II End-prep Enzyme Mix	0.75 μL	20×	1×

Prepare fresh immediately before use, keep on ice (0°C–4°C), and use within 1 h; do not store.

**Table:** Adapter ligation master mix (Step 85)

Reagent	Volume	Stock concentration	Final concentration
Native Adapter	5 μL	10×	1×
NEBNext Quick Ligation Reaction Buffer	10 μL	5×	1×
Quick T4 DNA Ligase	5 μL	10×	1×

Prepare fresh immediately before use, keep on ice (0°C–4°C), and use within 1 h; do not store.

## Step-by-step method details

### Total RNA isolation from tissues

**Timing: 1.5 h**

This section describes total RNA extraction from *Arabidopsis* seedlings, yielding intact RNA suitable for gene-specific reverse transcription.

1. Harvest ∼100 mg of *Arabidopsis* seedlings into a 2 mL tube with three 3-mm-diameter stainless steel beads and freeze using liquid nitrogen.
2. Grind the sample using TissueLyser II at 25 Hz for a total of 1 min (repeat 10 sec grind/10 sec freeze, six times).
3. Add 1 mL of NucleoZOL and mix vigorously by vortexing.

**CRITICAL:** NucleoZOL contains phenol and guanidinium salts. Handle it, and the isopropanol and ethanol used below, in a certified chemical fume hood while wearing appropriate personal protective equipment, and dispose of waste according to institutional guidelines.

***Note:*** Any extraction method that yields intact total RNA (e.g., TRIzol or silica-column kits) is compatible with this protocol; the NucleoZOL procedure above is the one used in Jeong et al.^1^

4. Incubate for 10 min at 22°C.
5. Centrifuge for 10 min at 12,000 × *g* at 22°C.
6. Transfer all supernatant to a fresh 2 mL tube.
7. Add 400 μL of nuclease-free DW, shake the sample vigorously for 15 sec, and incubate for 15 min.
8. Centrifuge for 15 min at 12,000 × *g* at 22°C.
9. Transfer 1 mL of supernatant to a fresh tube.
10. Add 1 mL of isopropanol and invert the sample to mix the solution.
11. Incubate for 10 min at 22°C.
12. Centrifuge for 10 min at 12,000 × *g* at 22°C.
13. Discard all supernatant using a pipette and add 1 mL of 75% ethanol.
14. Centrifuge for 5 min at 8,000 × *g.*
15. Discard all supernatant and spin down to collect all solution.
16. Discard all residual supernatant and add 200 μL of nuclease-free DW directly without a separate air-drying step.

**CRITICAL:** Do not air-dry the pellet. Residual ethanol is removed by thorough aspiration of the supernatant; omitting the drying step prevents overdrying, which would make the RNA difficult to redissolve.

***Note:*** Before proceeding, assess RNA quality by spectrophotometry (we recommend A260/280 ≈ 2.0 and A260/230 > 1.8). RNA integrity can be checked simply by intact rRNA bands on an agarose gel, or by RIN (Bioanalyzer/TapeStation) where available. Degraded or contaminated RNA reduces reverse-transcription efficiency and quantification reliability.

***Note:*** One microgram of total RNA was used and validated in this protocol; other inputs may require independent optimization. Store purified RNA at −80°C and avoid repeated freeze–thaw cycles.

### Synthesize cDNA with GSoligo(dT) to elongate GSP adapter to target transcript

**Timing: 20 min**

This section describes reverse transcription with the gene-specific oligo(dT) primer (GSoligo(dT)), which flanks each first-strand cDNA with the gene-specific sequence needed for single-primer pre-amplification.

17. Transfer 1 μg of total RNA to a fresh 200 μL PCR tube.
18. Add nuclease-free DW to a final volume of 8 μL.
19. Incubate the sample for 5 min at 65°C to denature the secondary structure of RNA.
20. Chill the sample immediately on ice for 5 min.
21. Spin down the sample.
22. Add 2 μL of gDNA wiper (from HiScript IV 1^st^ Strand cDNA Synthesis Kit) and mix gently.
23. Incubate the sample for 2 min at 42°C.
24. Prepare (sample number N + 1) of the RT master mix freshly and keep on ice.
25. Spin down the sample.
26. Add 10 μL of master mix to the sample and mix gently.
27. Incubate the sample for 5 min at 50°C for reverse transcription.
28. Sequentially incubate the sample for 10 sec at 85°C to inactivate RTase.
29. Directly use all products for the next step without purification.

***Note:*** Reverse-transcription incubations (Steps 19–28) are performed with the heated lid off, as the steps are short and the 65°C denaturation is followed immediately by chilling on ice.

**Pause point:** cDNA may be used immediately or stored at −20°C up to 1 week before pre-amplification; for longer storage keep at −80°C.

### Selective target pre-amplification for quantification

**Timing: 1.5 h**

This section describes limited-cycle pre-amplification with a single gene-specific primer (GSP) to enrich low-abundance target isoforms while preserving their relative abundances.

***Note:*** Limit pre-amplification to ≤12 cycles. In the validation dataset, *UBC21* Cq values began to decrease and additional melt-curve signal appeared beyond 12 cycles, consistent with non-specific amplification; 12 cycles was therefore used to preserve relative abundances (see expected outcomes and troubleshooting problem 3).

30. Prepare (N + 1) of the pre-amplification master mix freshly and keep on ice.

**CRITICAL:** Prepare the master mix to ensure an even PCR reaction across samples.

31. Add 30 μL of master mix to the sample.
32. Run PCR with the program below.

**Table:** 

Steps	Temperature	Time	Cycles
Initial denaturation	95°C	1 min	1
Denaturation	98°C	10 sec	≤12
Primer annealing	62°C	30 sec	
Extension	68°C	1 min/kb	
Final extension	72°C	10 min	1
Hold	4°C	∞	–

33. Spin down the PCR product and transfer to a fresh 1.5 mL tube.
34. Add 50 μL of nuclease-free DW and 70 μL of AMPure XP beads to 50 μL of PCR product.

***Note:*** — AMPure XP bead handling (applies to all bead cleanup steps below): Equilibrate the beads at 22°C for 30 min and resuspend them thoroughly by vortexing immediately before each use. Prepare fresh 80% ethanol on the day of use for all ethanol wash steps. Do not overdry the bead pellet before elution.

35. Mix the sample and beads by pulse vortexing 2–3 times.
36. Incubate the tube for 10 min at 22°C on an end-to-end rotor at 15 rpm.
37. Spin down the tube for 5 sec.
38. Pellet the beads on the magnetic rack for 5 min.
39. Discard all solution and wash the beads with 300 μL of freshly prepared 80% ethanol.
40. Repeat the previous wash once more.
41. Spin down the tube.
42. Pellet the beads on the magnetic rack and discard all residual ethanol.
43. Add 300 μL of nuclease-free DW to the tube and mix vigorously by vortexing.

***Optional:*** For nanopore sequencing, elute the sample with 10 μL of nuclease-free DW instead.

44. Incubate the tube for 10 min at 22°C on an end-to-end rotor at 15 rpm.
45. Spin down the tube and pellet the beads on the magnetic rack for 5 min.
46. Transfer the solution to a fresh 1.5 mL tube.

### Downstream quantification by qPCR analysis

**Timing: 1.5 h**

This section describes relative quantification of the pre-amplified isoforms by qPCR using isoform-specific primers and a validated reference gene.

47. Dilute each primer set to 1:20.

***Note:*** We recommend validating each isoform-specific qPCR primer set before routine use by standard-curve analysis on a serial dilution of pooled cDNA, confirming amplification efficiency (acceptable range 90%–110%), specificity (a single melt peak), sensitivity, and linearity (R^2^ > 0.98).

48. In the 96-well qPCR plate, add 8 μL of sample, 2 μL of primer mix, and 10 μL of qPCR master mix. Run each sample in technical triplicate.

***Note:*** For each RNA sample, carry a no-reverse-transcription (−RT) control through STALARD pre-amplification and qPCR to verify the absence of genomic-DNA contamination, and include a no-template control (NTC) on every plate to monitor contamination and primer–dimers.

49. Mix the plate vigorously and spin down.
50. Run qPCR on the Bio-Rad CFX96 with the program containing melt curve analysis.

**Table:** 

Steps	Temperature	Time	Cycles
Initial denaturation	95°C	2 min	1
Denaturation	95°C	20 sec	40
Primer annealing	60°C	20 sec	
Extension	72°C	20 sec	
Denaturation	95°C	10 sec	1
Gradual increment	60°C to 95°C, 0.5°C increment	5 sec	
Hold	4°C	∞	–

51. Analyze the relative transcript level using the 2−ΔΔCq method^4^ with a validated reference gene. *UBC21* was used in this protocol; users should validate reference-gene stability under their own experimental conditions.

**CRITICAL:** Inspect the melt curve for each reaction. A single sharp peak is consistent with a single predominant amplicon; additional peaks or a broad low-temperature peak suggest non-specific amplicons or primer–dimers, which confound the Cq. In that case, re-optimize the annealing temperature or redesign the primers before quantification (see troubleshooting problem 4).

### Nanopore sequencing library preparation

**Timing: 3 h**

This optional section describes preparation of a barcoded amplicon library from the pre-amplified (STALARD) product for molecule-level isoform quantification by nanopore sequencing.

#### Prepare cDNA

52. Transfer 130 ng of cDNA and adjust the volume to 12.5 μL using nuclease-free DW.

***Note:*** The STALARD product is double-stranded and is quantified with the Qubit dsDNA assay. Oxford Nanopore recommends ∼200 fmol of amplicon per sample for the end-prep/barcoding kit; 130 ng corresponds to ∼200 fmol for a ∼1 kb amplicon. Because STALARD amplicons vary in length, the mass required for a given molar amount should be calculated from the representative fragment length when strict molar normalization is needed. In this study, 130 ng was used.

***Optional:*** We recommend checking the cDNA concentration using the Qubit™ dsDNA Assay Kit.

#### End prep

53. Prepare (N + 1) of the end-prep master mix freshly and keep on ice.
54. Mix by pipetting and spin down.
55. Incubate for 5 min at 20°C and sequentially for 5 min at 65°C.
56. Transfer the sample to a fresh 1.5 mL tube.
57. Add 15 μL of resuspended AMPure XP beads to the sample and mix by flicking.
58. Incubate the tube for 5 min at 22°C on an end-to-end rotor at 15 rpm.
59. Spin down the tube and pellet the beads on the magnetic rack for 5 min.
60. Discard all solution and wash the beads with 200 μL of 80% ethanol.
61. Repeat the previous wash once more.
62. Spin down the tube.
63. Pellet the beads on the magnetic rack and discard all residual ethanol.
64. Add 10 μL of nuclease-free DW to the tube and mix vigorously by vortexing.
65. Incubate the tube for 10 min at 22°C on an end-to-end rotor at 15 rpm.
66. Spin down the tube and pellet the beads on the magnetic rack for 5 min.
67. Transfer the solution to a fresh 1.5 mL tube.

#### Barcode ligation

68. Transfer 7.5 μL of the sample to a fresh 200 μL PCR tube.
69. Add 2.5 μL of native barcode.
70. Add 10 μL of Blunt/TA Master Mix and mix by pipetting.
71. Incubate for 20 min at 22°C.
72. Add 4 μL of 0.5 M EDTA to the sample.
73. Pool all samples in a fresh 1.5 mL tube.
74. Add 0.7× volume of AMPure XP beads and mix by flicking.
75. Incubate for 10 min at 22°C on an end-to-end rotor at 15 rpm.
76. Spin down the tube and pellet the beads on the magnetic rack for 5 min.
77. Discard all solutions and wash the beads with 700 μL of 80% ethanol.
78. Repeat the previous wash once more.
79. Spin down the tube.
80. Pellet the beads on the magnetic rack and discard all residual ethanol.
81. Add 35 μL of nuclease-free DW to the tube and mix vigorously by vortexing.
82. Incubate for 10 min at 37°C (agitate the sample by gentle flicking every 2 min).
83. Spin down the tube and pellet the beads on the magnetic rack for 5 min.
84. Transfer the solution to a fresh 1.5 mL tube.

#### Adapter ligation

85. Prepare (N + 1) of the adapter ligation master mix freshly and keep on ice.
86. Transfer 30 μL of the pooled barcoded sample to a fresh 200 μL PCR tube.
87. Add 20 μL of master mix and mix by pipetting.
88. Incubate for 20 min at 22°C.
89. Add 20 μL of AMPure XP beads and mix by flicking.
90. Incubate for 10 min at 22°C on an end-to-end rotor at 15 rpm.
91. Spin down the tube and pellet the beads on the magnetic rack for 5 min.
92. Discard all solution and wash the beads with 125 μL of Short Fragment Buffer.
93. Repeat the previous wash once more.
94. Spin down the tube.
95. Pellet the beads on the magnetic rack and discard all residual Short Fragment Buffer.
96. Add 15 μL of elution buffer to the tube and mix vigorously by vortexing.
97. Incubate for 10 min at 37°C (agitate the sample by gentle flicking every 2 min).
98. Spin down the tube and pellet the beads on the magnetic rack for 5 min.
99. Transfer the solution to a fresh 1.5 mL tube.
100. Run sequencing according to the manufacturer’s instructions.

***Note:*** Before loading, quantify the final barcoded library using Qubit dsDNA assay and, where possible, confirm the expected fragment-size distribution by TapeStation/Bioanalyzer or gel. Load the recommended mass onto the flow cell.

## Expected outcomes

**Figure 1:**
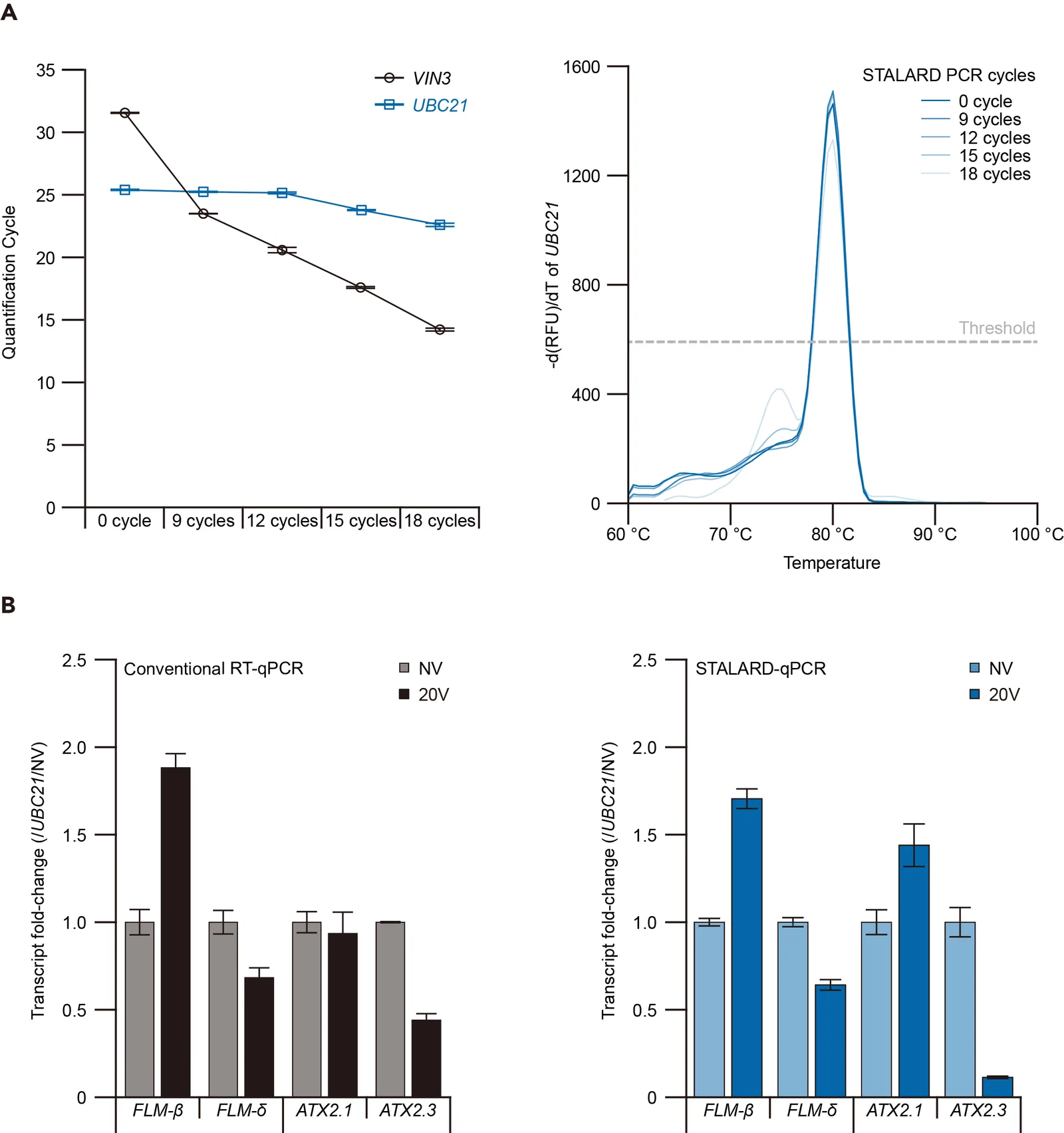
Validation of STALARD (A) Cq values before and after STALARD pre-amplification of the low-abundance gene *VIN3* and the reference gene *UBC21*, and melt peak analysis of *UBC21* after STALARD. Data represent the mean ± SD from three technical replicates. (B) Detection of alternative splicing pattern changes in *FLM* and *ATX2* before and after vernalization treatment using STALARD. Transcript levels are normalized to NV and shown as mean ±  standard error of the mean (SEM) from three biological replicates. Figure adapted from Jeong et al.^1^ with permission.

STALARD pre-amplification should yield a marked reduction in Cq values for low-abundance transcripts. For instance, *VIN3* under non-vernalized conditions, which is typically detected at Cq 31.55 by conventional RT-qPCR, shifts to Cq 20.58 after 12 cycles of STALARD pre-amplification from 1 μg input RNA (Figure 1A). This improvement of 10–12 cycles brings the target into the reliable quantification range. High-abundance reference gene *UBC21* remains stable across amplification cycles up to 12, confirming that enrichment is selective for low-abundance transcripts. In addition, melt peak analysis of the high-abundance reference *UBC21* after STALARD up to 12 cycles shows a single sharp peak, confirming that amplification within the recommended cycle range does not generate non-specific byproducts (Figure 1A). Together, these outcomes demonstrate that the protocol enables reliable and reproducible quantification of transcripts that are otherwise beyond the detection limit of conventional RT-qPCR.

Furthermore, the application of STALARD enabled accurate quantification of isoform expression changes in response to environmental conditions. For example, in *FLM*, the *FLM-β* isoform increased, and the *FLM-δ* isoform decreased after cold treatment. This pattern was consistently observed by both conventional RT-qPCR and STALARD-qPCR, in agreement with previous reports (Figure 1B). By contrast, in *ATX2*, isoform-specific changes—an increase in *ATX2.1* and a decrease in *ATX2.3* after cold treatment—could not be reliably quantified by conventional RT-qPCR due to low abundance but were faithfully reproduced using STALARD-qPCR (Figure 1B). These results confirm that STALARD facilitates accurate quantification of isoforms at or near the lower limit of quantification (LLOQ). Here, the LLOQ is defined as the lowest input concentration that can be reliably quantified while meeting specified criteria for accuracy and precision within the assay.^2^ This enables reliable detection of biologically meaningful expression changes that conventional RT-qPCR often fails to resolve.

## Limitations

STALARD has several important limitations that should be considered before application. First, the protocol requires prior knowledge of the 5′-end sequence of the target transcript, which is essential for primer design. Isoforms that originate from alternative transcription start sites or carry truncated 5′ ends will not be detected, as they lack the defined sequence needed for amplification. Second, because STALARD relies on an oligo(dT)-based priming strategy, its application is restricted to polyadenylated RNAs. Non-polyadenylated transcripts, including many non-coding RNAs, are therefore not captured. Third, over-amplification beyond 12 PCR cycles can introduce non-specific products and distort isoform ratios, making careful control of cycle number critical.^5^^,^^6^ Fourth, the performance of STALARD depends strongly on primer design quality; poorly designed primers may fail to enrich low-abundance targets or may introduce amplification bias. In addition, protocol performance depends on input RNA quality. Because quantification relies on reverse transcription, degraded or inhibitor-contaminated RNA lowers reverse-transcription efficiency and can compromise quantification accuracy, particularly for the lowest-abundance targets; assessing RNA integrity and purity before starting is therefore recommended (see Step 16). Finally, as a targeted approach, STALARD is not intended to replace transcriptome-wide profiling methods such as RNA-seq, but rather to complement them by enabling sensitive and accurate quantification of selected isoforms.

Optional extension (outside the scope of this protocol): For applications where the efficiencies of reverse transcription and STALARD pre-amplification are expected to differ substantially between samples (e.g., markedly different RNA integrity or strong inhibitors), users may add an external RNA spike-in prior to reverse transcription as an internal control for the combined RT–pre-amplification efficiency, which a reference gene alone does not capture. To traverse the same workflow as endogenous targets, the spike-in should share the 5′ GSP sequence and carry a poly(A) tail (e.g., 5′-GSP–non-homologous unique sequence–A50-3′), with the internal sequence quantified by a dedicated primer set. A fixed amount is added to equal input RNA per sample, and its recovered signal is used to normalize target measurements. Implementation requires independent optimization and validation: (i) titrating the spike-in amount to fall within the targets’ quantifiable range, (ii) confirming primer specificity to the unique sequence, and (iii) verifying no competition with endogenous targets. These parameters are assay-dependent and are therefore not detailed here.

The STALARD product is compatible with nanopore amplicon sequencing (see optional: nanopore sequencing library preparation). However, rigorous inter-sample quantitative comparison based on nanopore read counts depends on additional factors beyond STALARD itself (e.g., library preparation efficiency, sequencing depth, and barcode balance) and has not been benchmarked in this protocol. Users should validate quantitative behavior in their own experimental context.

## Troubleshooting

### Problem 1

No amplification of the target transcript (related to Steps 30–46).

RNA degradation or an incorrectly designed gene-specific primer (GSP).

### Potential solution

• Verify RNA integrity by gel electrophoresis or Bioanalyzer, and check purity (A260/280 ≈ 2.0, A260/230 > 1.8), before reverse transcription.
• Redesign the GSP using a 20–25 bp sequence at the 5′-end with Tm 60°C–62°C and minimal secondary structure.

### Problem 2

Non-specific amplification or primer–dimers (related to Steps 30–46).

Too many PCR cycles (>12) or primers with hairpin/dimer potential.

### Potential solution

• Reduce PCR cycles to ≤12.
• Raise the annealing temperature slightly (62°C–64°C).
• Re-check primer design using software such as Primer3.

### Problem 3

Reference gene Cq values decrease after pre-amplification (related to Steps 30–51).

Over-amplification leading to enrichment of abundant transcripts.

### Potential solution

• Limit STALARD cycles to ≤12.
• Always include a high-abundance control (e.g., *UBC21*) to confirm that selectivity remains intact.

### Problem 4

qPCR melt curve shows additional peaks after STALARD pre-amplification (related to Steps 47–51).

Non-specific amplification products or primer–dimers are generated when the PCR cycle number exceeds 12, or when primer design allows secondary binding.

### Potential solution

• Limit the number of pre-amplification cycles to ≤12.
• Optimize annealing temperature (62°C–64°C).
• Confirm primer specificity in silico before synthesis, and experimentally by a single melt-curve peak (or amplicon-size check on a gel).
• If an additional peak is also present in the NTC, optimize or redesign the qPCR primers. If it appears only after STALARD pre-amplification, inspect the pre-amplified product by gel electrophoresis, reduce the pre-amplification cycle number, and re-evaluate the GSP and annealing conditions.

### Problem 5

Poor quantification linearity or inconsistent fold-changes across samples (related to Steps 47–51).

Operation outside the linear range (near the LLOQ) or suboptimal primer performance can bias 2^−ΔΔCq^ estimates.

### Potential solution

• Validate each assay on a pooled cDNA dilution series; confirm efficiency (90%–110%) and linearity (R^2^ > 0.98), and define the assay-specific LLOQ using predefined criteria for quantitative accuracy and precision.
• Keep inputs within the validated linear range and use technical replicates.

## Resource availability

### Lead contact

Further information and requests for resources and reagents should be directed to and will be fulfilled by the lead contact, Ilha Lee (ilhalee@snu.ac.kr).

### Technical contact

Technical questions on executing this protocol should be directed to and will be answered by the technical contact, Daesong Jeong (tiger817@snu.ac.kr).

### Materials availability

This study did not generate new unique reagents. All primers described in this protocol are user-defined custom synthesis products (representative sequences are listed in the key resources table).

### Data and code availability

All data presented in Figure 1 are derived from the same experiments as reported in Jeong et al.^1^ The published article includes all data analyzed during this study. This study did not generate or analyze new code.

## Acknowledgments

We thank members of the Laboratory of Plant Developmental Genetics for helpful discussions. This work was supported by the 10.13039/501100003725National Research Foundation (NRF) of Korea (RS-2021-NR060084 and RS-2025-00520643).

## Author contributions

D.J. performed the experiments and analyzed the data. C.P. contributed to data analysis and visualization. I.L. conceived and supervised the study and wrote the manuscript with input from all authors.

## Declaration of interests

D.J. and I.L. are inventors on a patent application filed by the Seoul National University R&DB Foundation related to the STALARD method described in this protocol.
